# Absolute Spatially- and Temporally-Resolved Optical Emission Measurements of rf Glow Discharges in Argon

**DOI:** 10.6028/jres.098.012

**Published:** 1993

**Authors:** S. Djurović, J. R. Roberts, M. A. Sobolewski, J. K. Olthoff

**Affiliations:** National Institute of Standards and Technology, Gaithersburg, MD 20899-0001

**Keywords:** argon, discharge, gaseous electronics, optical emission, plasma, rf, spatial profile, temporal dependence

## Abstract

Spatially- and temporally-resolved measurements of optical emission intensities are presented from rf discharges in argon over a wide range of pressures (6.7 to 133 Pa) and applied rf voltages (75 to 200 V). Results of measurements of emission intensities are presented for both an atomic transition (Ar I, 750.4 nm) and an ionic transition (Ar II, 434.8 nm). The absolute scale of these optical emissions has been determined by comparison with the optical emission from a calibrated standard lamp. All measurements were made in a well-defined rf reactor. They provide detailed characterization of local time-resolved plasma conditions suitable for the comparison with results from other experiments and theoretical models. These measurements represent a new level of detail in diagnostic measurements of rf plasmas, and provide insight into the electron transport properties of rf discharges.

## 1. Introduction

A fuller understanding of the rf glow discharges utilized for the etching of semiconductor materials requires knowledge of the microscopic gas-phase collision processes that occur in the plasma. Optical emission spectroscopy is a non-intrusive technique that allows the detection of atoms, molecules, and ions in excited states within the plasma. Analysis of the spatial and temporal distributions of the optical emission from an rf glow discharge also provides information concerning electron transport properties and the electron-energy distributions in the plasma.

The number of studies investigating the time-varying optical emission from rf plasmas at frequencies near 13.56 MHz are limited. De Rosny et al. [1] have observed the time-dependent emission from a short-lived state of Si in a pure SiH_4_ rf plasma. Donnelly and coworkers [2] reported the effects of frequency on the time-varying optical emission from chlorine-containing rf discharges. Bletzinger and De Joseph [3] investigated the temporally-resolved optical emission from a nitrogen plasma as a function of position between the rf electrodes. More recently Tochikubo et al. [4] and Köhler et al. [5] reported spatially- and temporally-resolved optical emission data for neutral and ionic transitions that occur in argon plasmas. Tochikubo et al. [4] calculated the relative net excitation rates by deconvoluting the optical emission profile, while Köhler et al. [5] performed Boltzmann calculations to derive excited state populations for comparison with the time-modulated emission intensity.

Recent modeling of low-pressure rf glow discharges has begun to predict many of the time-varying aspects of high-frequency rf plasmas [6–10]. However, the presently available time-resolved optical emission data is insufficient to fully validate the conclusions derived by plasma modeling; either because the data do not cover an adequate range of experimental conditions or because the experimental conditions are not adequately defined. Additionally, the time-resolved optical emission data presently in the literature consist only of relative emission intensities which do not allow for comparison with calculations of absolute excitation rates or absolute population densities.

The emphasis of the present work is therefore to measure the absolute, time-resolved and spatially-resolved optical emission intensities from “well-defined” argon plasmas over a wide range of pressures and voltages. All experiments were performed on a Gaseous Electronics Conference (GEC) rf reference cell [11,12]. Ostensibly identical copies of this cell have been assembled and characterized in several laboratories. For each rf voltage and pressure setting, the time-varying optical emission intensity was measured absolutely for an Ar I (750.4 nm) and Ar II (434.8 nm) line as a function of observation location along the central axis between the parallel-plate electrodes by using a calibrated monochromator. No radial emission profiles were taken. Voltage and current waveforms were also measured for each set of plasma conditions, and analyzed to determine the time-dependent voltage and current signals at the surface of the powered electrode in contact with the plasma.

## 2. Experimental Details

### 2.1 GEC rf Reference Cell

All experiments were performed using a GEC rf reference cell. The GEC rf reference cell is an rf plasma research reactor whose design was initiated at the 1988 Gaseous Electronics Conference (Oct. 18–22, Minneapolis, MN). The reference cell was designed to provide a standard experimental platform for rf plasma research that was physically identical from laboratory to laboratory, so that experimental results, theoretical models, and various plasma diagnostics could be more easily compared.

The details of the design of the GEC rf reference cell are given in Refs. [11] and [12]. Briefly, the cell is a parallel-plate discharge chamber with 102 mm diameter electrodes separated by 25 mm. The electrodes are cylindrically symmetric and their surfaces are horizontal. The top electrode contains 169 holes (380 *μ*m diameter) to provide a showerhead gas inlet and is grounded to the outer wall of the vacuum chamber. The bottom electrode is powered by an ENI^2^ 13.56 MHz rf power supply isolated with a 0.1 *μ*F blocking capacitor. For the data presented here, flow rates were 20 standard cubic centimeters per minute (sccm), pressures were varied from 6.7 to 133.3 Pa, and peak-to-peak rf voltages ranged from 75–200 V. The cylindrical vacuum chamber is constructed of stainless steel and has eight radial copper-gasket flanges at the chamber midplane. Two 203 mm diameter flanges are fitted with 136 mm diameter quartz windows for spectroscopic observations. Two 152 mm flanges are orthogonal to these, one of which accommodates a turbomolecular pump for establishing a base pressure of <10^−5^ Pa. Four 70 mm diameter flanges at 45° with respect to the four larger flanges are also mounted at the cell mid-plane. The bottom of the vacuum chamber is constructed so the pumpout of the gas is accomplished by four symmetrically placed 70 mm diameter ports piped into a single exhaust line to a mechanical vacuum pump.

**Fig. 1:**
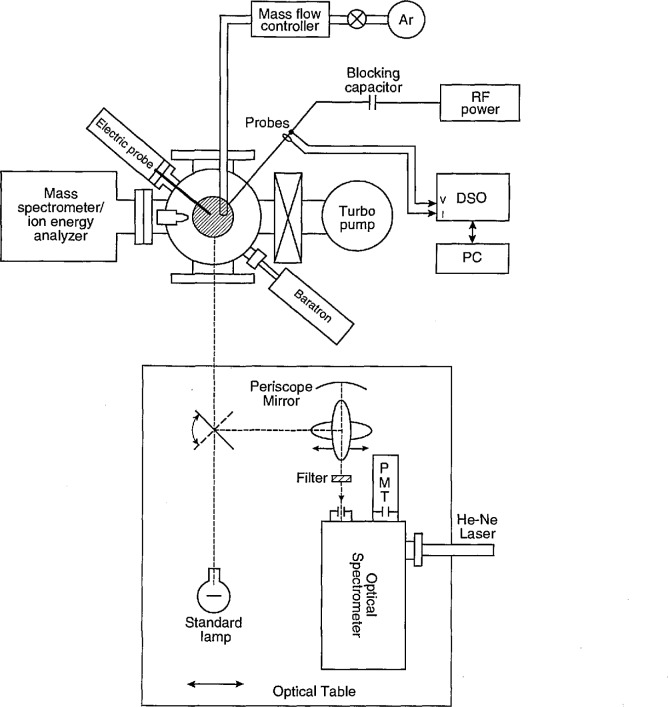
Diagram of the NIST GEC rf reference cell, optical setup, and other diagnostics; where PMT is the photomuliplier tube, DSO is the digital storage oscilloscope, and PC is a personal computer. The mass spectrometer/ion energy analyzer and electrical probe are discussed elsewhere [13–15].

On the GEC rf reference cell at NIST, a mass spectrometer with an ion energy analyzer has been mounted on one of the 152 mm diameter side ports and an electrical plasma probe has been attached to one of the 70 mm diameter ports (see Fig. 1). Detailed discussions of these diagnostics are presented elsewhere [13–15].

### 2.2 Electrical Measurements

Voltage and current waveforms were measured on the powered electrode lead using a voltage probe and a Pearson current probe with upper 3 dB frequencies of 300 and 200 MHz, respectively. The probe signals were digitized by a Phillips PM3323 oscilloscope and then transferred to a computer for Fourier analysis. To extract the amplitude and phase of the significant Fourier components, an iterative least squares curve fitting algorithm was used instead of discrete Fourier transform techniques, which only provide Fourier coefficients at frequencies commensurate with the sampling frequency. The curve-fitting algorithm, which operates very efficiently, is described elsewhere [16]. The fits included a dc component, the fundamental (13.56 MHz) component and the second through fifth harmonics. The signals at the harmonic frequencies, which arise because the impedance of the plasma is nonlinear, contain additional information not present in the fundamental components.

Phases obtained from the fits were corrected to account for propagation delays in the cables connecting the probes to the oscilloscope. Current and voltage values were subsequently corrected for the effects of stray impedance. Due to the stray impedance of the cell, the current and voltage waveforms at the point outside the cell where the probes are located, *I_m_*(*t*) and *V_m_*(*t*), will differ from the waveforms actually present at the powered electrode, *I_p_*(*t*) and *V_P_*(*t*). For example, some of the measured current *I_m_*(*t*) is drawn not by the plasma but by parasitic capacitances in the electrode assembly and in the probes. Also, *V_m_*(*t*) will differ from *V_P_*(*t*) due to a voltage drop along the power lead, as its inductance is significant at radio frequencies. To correct for these effects we use a technique described previously [11]. A set of measurements made in the absence of a plasma fully determines the cascade matrix of the network of parasitics between the measurement point and the surface of the powered electrode. This matrix then serves to convert the waveforms measured when plasma is present, *I_m_*(*t*) and *V_m_*,(*t*), into the waveforms *I_p_*(*t*) and *V_p_*(*t*).

When parasitic currents are large, the corrected values are extremely sensitive to small errors in the measured values [17]. To avoid this problem, a shunt circuit consisting of a coil and an air variable capacitor, was connected between the power lead and the chamber ground at a point between the current probe and the chamber [11]. The shunt is designed to have a net inductive impedance at 13.56 MHz that will cancel, at that frequency, the net capacitive reactance of parasitics in the cell. The variable capacitor allows fine tuning of the impedance of the shunt and also maintains capacitive coupling to the cell so as not to short out the dc bias of the powered electrode. With the cell evacuated below 10^−5^ Pa and excited at 13.56 MHz, the capacitor was adjusted until the measured current was nulled. When nulled, the cell still draws parasitic current, but the shunt draws an equal and opposite current. Later, when gas is introduced and a plasma is generated, this is still approximately true; the current drawn by cell parasitics is still roughly canceled by the current drawn by the shunt. Excluding the large parasitic currents from the measurement branch results in increased precision, and also alleviates some concerns about rf interference and current probe overload. The characterization of cell parasitics is performed with the shunt in place and tuned, so that the correction for the effects of the parasitics includes the effects of the shunt as well.

### 2.3 Optical Emission Measurement System

The experimental setup for spectroscopic measurements is shown schematically in Fig. 1. The spectroscopic apparatus consists of a 2/3 m Czerny-Turner type grating monochromator with a 1200 lines/mm grating. This monochromator is equipped with a cooled Burle C31034A photomultiplier for pulse detection of the optical emission signal. The monochromator is equipped with a retractable mirror near its exit slit so a He-Ne laser may be substituted for the detector for alignment purposes. The vertical entrance and exit slits of the monochromator are typically 100 *μ*m wide and 2 mm high. The slit widths and photomultiplier voltage were kept constant (100 *μ*m and 1700 V, respectively) for all measurements, and metallic film neutral density filters were used, when necessary, to reduce the photon signals to suitable count rates for the pulse counting electronics used for data acquisition. Calibration of these filters is discussed in the next section.

The optics to image the plasma onto the monochromator slit are front surface mirrors with coatings to efficiently reflect the plasma emission at wavelengths from 200 nm to 1500 nm. There are three flat mirrors and one concave mirror, each 152 mm in diameter. The concave mirror (650 mm focal length) is positioned so that the plasma image is demagnified onto the entrance slit by approximately a factor of 2. These mirrors are arranged to act as a periscope bringing the level of the plasma emission to the same height as the monochromator, as well as rotating the image of the plasma by 90°. By this rotation, the electrode surfaces are imaged parallel to the long dimension of the entrance slit, thus permitting observations close to the electrode surface and providing higher spatial resolution of the plasma along the vertical axis. The spatial resolution was 0.5 mm vertically and 4 mm horizontally. Because of the periscope, scanning of the plasma emission between the electrodes can be accomplished by translating one of the mirrors (see Fig. 1).

### 2.4 Calibration of the Optical System

A tungsten ribbon filament lamp calibrated for spectral radiance (±2%) by the Radiometric Physics Division, NIST [18] is mounted on the optics table and is substituted for the plasma source by rotating one of the flat mirrors to image the lamp filament onto the slit (see Fig. 1). This lamp is used to calibrate the optics/monochromator system to obtain absolute spectral radiance measurements. The calibration measurements were made with the same slit width and photomultiplier voltage as used in the plasma measurements. The standard lamp had to be mounted with the long dimension of its filament in a horizontal position to accommodate the 90° rotation of the optical system. This is not a conventional configuration for a radiance calibrated standard lamp; therefore an estimate of the effects of this configuration had to be made. It was determined from a separate experiment that this rotation amounted to an additional uncertainty of ± 2% in the absolute radiance calibration at the wavelengths of the observed spectral lines.

An additional uncertainty in the calibration procedure arose because the intensity of the standard lamp signal was stronger than the observed spectral lines by approximately a factor of 10^3^. To prevent the measurement from saturating, metallic film neutral density filters were placed in the optical path in front of the monochromator slit for measurement of the lamp signals. For measurements at longer wavelengths, second order radiation from the lamp had to be blocked. On the other hand, for measurements at shorter wavelengths, the strong lamp radiation at longer wavelengths also had to be blocked to avoid a significant contribution to the signal from scattered light. Bandpass interference filters were incorporated into the optical path to serve both of these requirements. The bandpass filters were used only in the standard lamp calibration procedure, and not in the plasma experiments. Interference filters were used in the above procedure, since the use of color glass filters to perform the same functions created additional problems due to their fluorescence at certain wavelengths and changing transmission characteristics as a function of incident light flux. The metallic film neutral density filters and the bandpass filters could not be characterized using this optical monochromator system, and were therefore individually calibrated for transmission over the wavelength region of interest by a double monochromator system at NIST used for spectral radiance calibrations. The uncertainty associated with the calibration of these filters is estimated to be ±5%. The total uncertainty (1*σ*) in the measurement of a spectral line radiance is therefore estimated to be *±* 6%.

### 2.5 Time-Resolved Optical Detection System

**Fig. 2:**
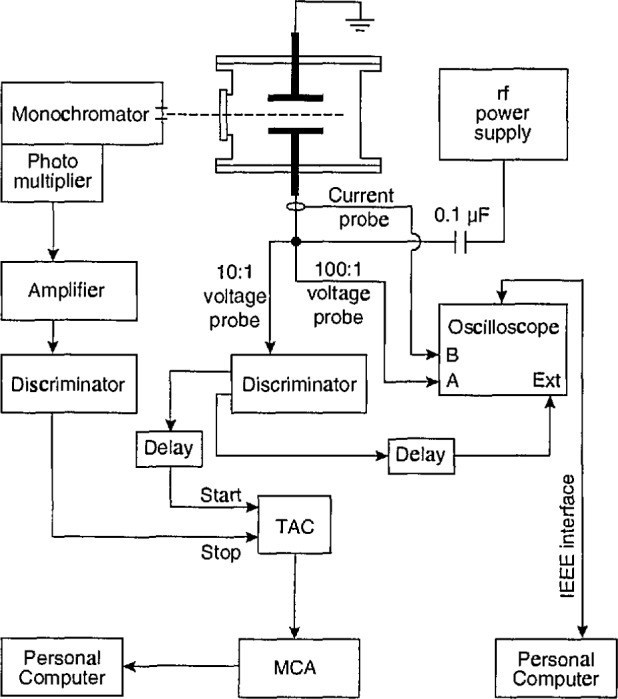
Schematic diagram of the timing electronics utilized to measure the temporal variation of the optical emission signal from argon plasmas for comparison with the applied rf voltage waveform. The TAC is a time-to-amplitude converter, the MCA is a multichannel analyzer, and Ext refers to the external trigger of the digital storage oscilloscope.

The time-varying output from the photomulti-plier was recorded with a system utilizing a time-to-amplitude converter (TAC) and a multi-channel analyzer (MCA). A detailed schematic diagram is shown in Fig. 2. The timing cycle of the TAC is initiated by a fast pulse derived by a 200 MHz discriminator from a 10:1 voltage probe attached to the bottom of the powered electrode. The timing cycle is stopped by a photon-initiated pulse from the photomultiplier system, or by the ending of the preset TAC timing period. For the data presented here, the maximum time measured by the TAC was set to 200 ns (slightly less than 3 rf cycles). As mentioned previously, photon count rates were kept low (< 10^5^ counts/s) by placing calibrated neutral density filters in the optical path for the measurement of the most intense optical signals. Because much less than one photon is detected, on the average, for each timing cycle, there is no discrimination against photons detected later in the timing cycle.

The output pulses from the TAC, whose magnitudes are proportional to the time between the trigger from the voltage waveform and the detection of the photon, are sorted into channels by the MCA. This accumulated spectrum represents the time-resolved optical emission signal for a single spatial location. Observation times at each position were 3 min for the Ar I line and 5 min for the less intense Ar II line. Measurements were made at 10–15 different positions between the electrodes, depending upon the degree of spatial variation in the signal intensity.

The MCA was set to record 256 channels providing a timing resolution of 0.78 ns/channel (200 ns/256 channels). More channels could have been utilized, thereby providing a smaller time increment. However, the uncertainty in timing measurements (discussed in the next section) and the broad distribution in time of the optical signal eliminate any inherent advantage to a finer time increment. Also, using more channels in the MCA would have significantly increased the data-taking time at each observation position.

### 2.6 Calibration of Timing Circuit

In order to correlate the time-varying optical emission signal with the applied rf voltage, it is necessary to calibrate the time delays of the entire optical/detector/electronics system. The voltage waveform is actually measured approximately 20 cm from the bottom of the powered electrode. The transit time from this point to the electrode surface is ~0.3 ns; which is nearly insignificant. In contrast, the time delays inherent in the voltage probe, cables, discriminator, amplifier, TAC, MCA, and oscilloscope are not insignificant, and were determined by simultaneously applying fast pulses to the external trigger of the dual trace digital oscilloscope, to the 100:1 voltage probe attached to oscilloscope channel A, to the start of the TAC, and to the input cable of the amplifier following the photomultiplier. The time delay units in the electrical circuit were then adjusted so that signals on the scope and MCA both appeared in the zero channel.

The time delay between the emission signal and the output of the photomultiplier was determined in the following way. A fast avalanche photo-diode, the 300 MHz oscilloscope, and a fast pulse generator were used to calibrate the delay time of a pulsed diode laser (Hamamatsu Picosecond Light Puiser). This laser was then positioned near the standard lamp at the focal point of the optical system. The time delay between triggering the pulsed laser and detection of the output pulse from the photomultiplier was measured using the oscilloscope and fast pulse generator. The time delay in the optical system (adjusted for the time delay of the laser) was 47 ns and was accounted for by adding this time to the time delay unit located prior to the start input of the TAC. The estimated uncertainty in the timing measurements is ±3 ns, and is primarily due to the finite rise time (~2 ns) of the fast electrical pulses used for calibration.

## 3. Results

The time-dependent emission intensities of a neutral argon line (750.4 nm) and an ionic argon line (434.8 nm) were measured at each observation point between the electrodes. The upper level of the Ar I transition has an atomic lifetime of approximately 20 ns [19] while the upper level of the Ar II transition has an atomic lifetime of approximately 7 ns [19]. These lines were chosen for study because: 1) they are well separated from possible interfering lines; 2) they are of sufficient intensity to allow for reasonable data acquisition times; 3) they have atomic lifetimes that are less than the 13.56 MHz period (73.7 ns); and 4) some previously published data exists in the literature for comparison [4, 5].

**Fig. 3:**
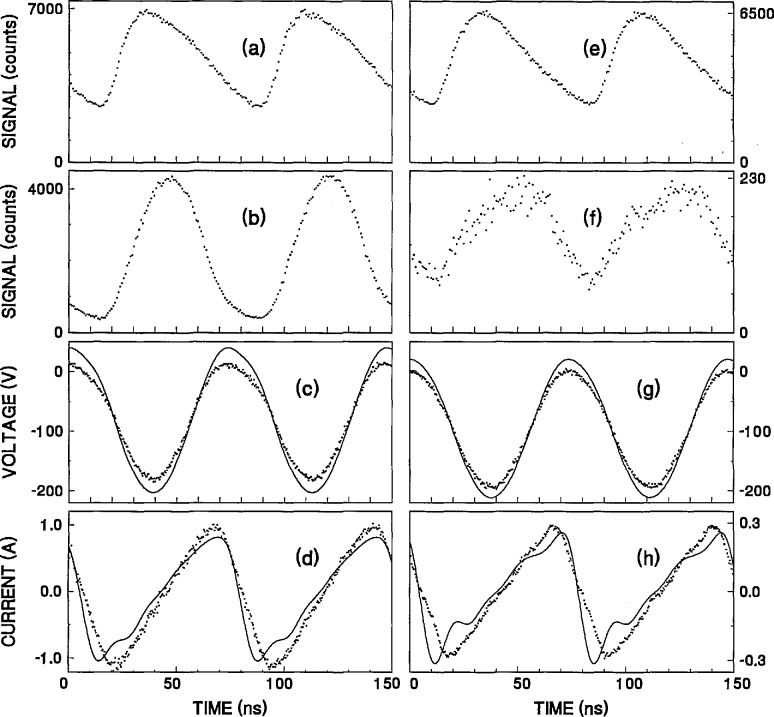
Measured time-dependent optical emission signals, and the corresponding voltage and current waveforms from argon plasmas with applied rf voltages of 200 V and pressures of 133.3 Pa (a–d) and 13.3 Pa (e–h). (a) noncalibrated optical emission Ar I signal at 750.4 nm from an observation point 21 mm from the grounded electrode; (b) noncalibrated Ar II optical signal at 434.8 nm from an observation point 22.5 mm from the grounded electrode; (c) ⋯ measured voltage waveform obtained simultaneously with the optical emission data in 3a and 3b, — calculated voltage waveform at the surface of the powered electrode; (d) ⋯ measured current waveform corresponding to the voltage waveforms in 3c, — calculated current waveform at the surface of powered electrode; (e) noncalibrated optical emission Ar I signal at 750.4 nm from an observation point 17 mm from the surface of the grounded electrode; (f) noncalibrated Ar II signal at 434.8 nm from an observation point 18.5 mm from the grounded electrode; (g) ⋯ measured voltage waveform obtained simultaneously with the optical emission data in 3e and 3f, — calculated voltage waveform at the surface of the powered electrode; (h) ⋯ measured current waveform corresponding to the voltage waveform in 3g, — calculated current waveform at the surface of the powered electrode.

Examples of correlations between the measured time-dependent optical emission signals and the voltage and current waveforms are shown in Fig. 3 for argon plasmas with uncorrected peak-to-peak rf voltages of 200 V and gas pressures of 133.3 Pa (Figs. 3a–3d) and 13.3 Pa (Figs. 3e–3h). Figure 3a shows the raw Ar I optical emission signal as recorded by the MCA at a point where the emission signal was a maximum (21 mm from the grounded electrode). The observation time was 3 min and a filter with 0.6% transmission was used to reduce the count rate to acceptable levels. The raw data for the Ar II line are shown in Fig. 3b for the same plasma at an observation point 22.5 mm from the grounded electrode (also the location of the maximum emission intensity). The observation time was 5 min and no transmission filters were used. The dotted curves in Fig. 3c and 3d show the voltage and current waveforms, *V_m_*(*t*) and *I_m_*(*t*) acquired simultaneously by the digital oscilloscope with the data shown in Figs. 3a and 3b. The solid curves represent the voltage and current waveforms, *V_p_*(*t*) and *I_P_*(*t*), at the surface of the powered electrode as calculated by the equivalent circuit model discussed in Sec. 2.2. The presence of higher harmonics in the electrical waveforms is indicated by the nonsinusoidal character of the curves.

Similar data obtained for a lower pressure plasma (13.3 Pa) are shown in Figs. 3e–3h. The Ar I optical emission data in Fig. 3e were obtained at a point 17 mm from the surface of the grounded electrode with a 2.9% transmission filter. The Ar II data in Fig. 3f were obtained at a point 18.5 mm from grounded electrode, and no transmission filters were used.

The optical emission profiles for the Ar I and Ar II lines, as shown in Fig. 3, are quite different. For the plasma at 133.3 Pa, the peaks in the time-varying Ar I optical emission signal are broader than in the Ar II profile and exhibit an assymmetry on the right hand side of the temporal peaks. These characteristics are due to the relatively long atomic lifetime (20 ns) of the neutral excited state. This also contributes to the significant “background” or non-time-dependent portion of the Ar I signal observed in Fig. 3a. For other transitions whose excited-state lifetimes are even longer (approaching the rf period), the fraction of the optical signal exhibiting a time-dependence is very small due to the “smearing-out” effect of the long atomic lifetimes [5]. In contrast, the Ar II signal profile at 133.3 Pa exhibits narrow temporal peaks and a large time-dependent signal due to the short lifetime of the excited state (~7 ns) as compared with the rf period. At 13.3 Pa, the Ar I profile is very similar to that observed at 133.3 Pa, however, the peaks in the Ar II profiles are much broader than at higher pressures. This indicates a change in the excitation of the Ar II transition as the pressure changes.

Comparison of the optical-emission data in Fig. 3 also indicates that the Ar I and Ar II profiles are not in phase with each other, with the Ar I profile leading the Ar II profile. The Ar II time-dependence at 133.3 Pa is observed to be nearly 180° out-of-phase with the applied voltage, as was previously reported by Tochikubo et al. [4]. Due to the non-sinusoidal nature of the current waveform, it is difficult to draw correlations between the current and the optical emission signals.

**Fig. 4:**
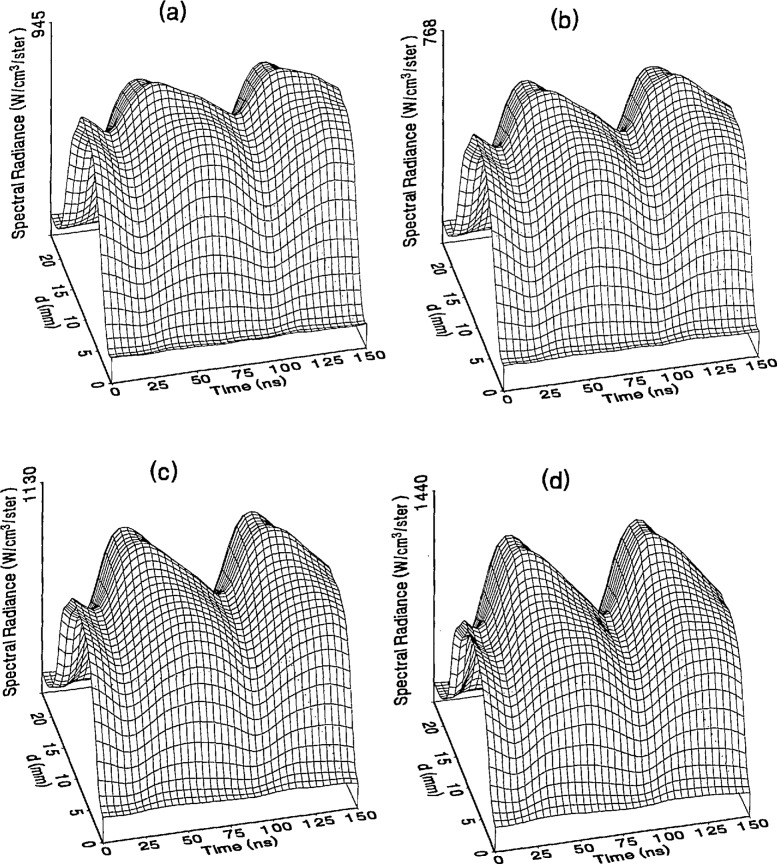
Optieal emission measurements of the spatial profile and temporal evolution of the Ar I 750.4 nm emission line from a 6.7 Pa argon plasma at (a) 75 V, (b) 100 V, (e) 150 V, and (d) 200 V applied rf voltages.

**Fig. 5:**
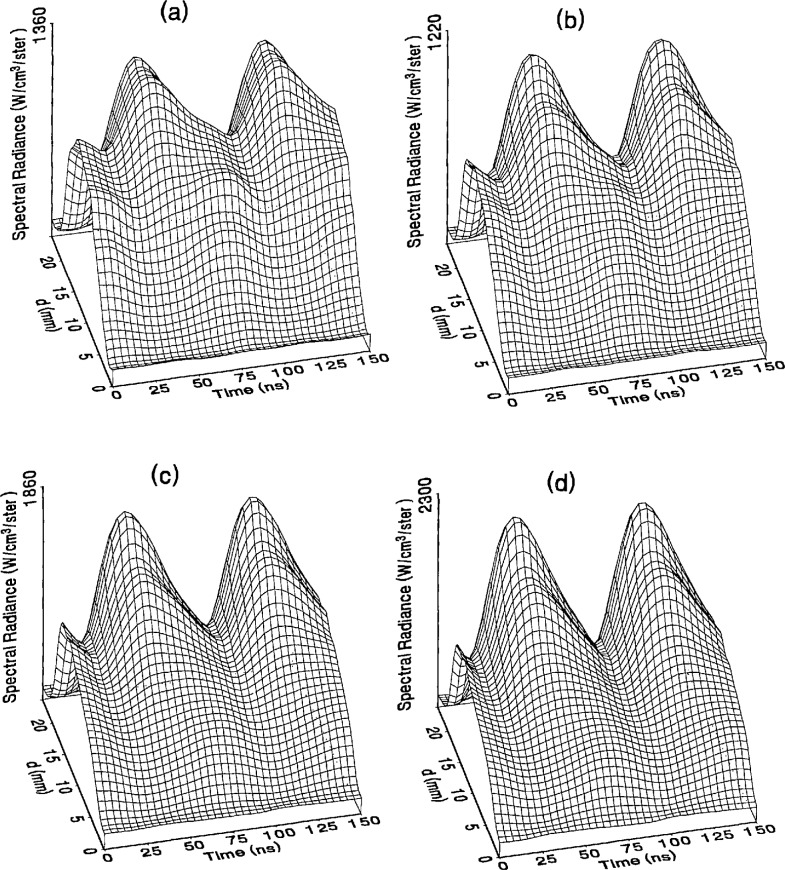
Optical emission measurements of the spatial profile and temporal evolution of the Ar I 750.4 nm emission line from a 13.3 Pa argon plasma at (a) 75 V, (b) 100 V, (c) 150 V, and (d) 200 V applied rf voltages.

**Fig. 6:**
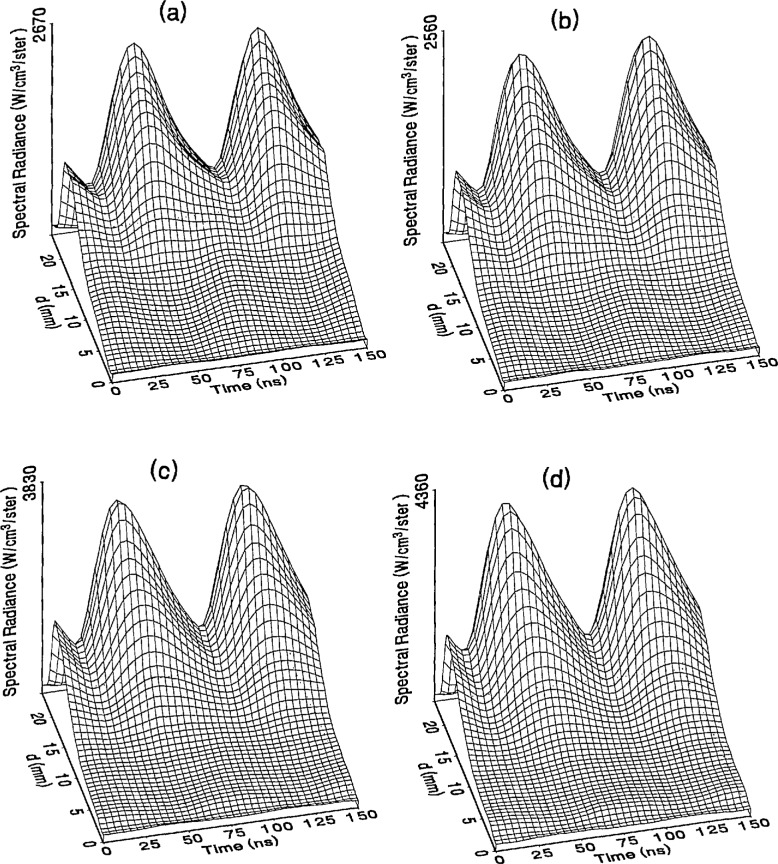
Optical emission measurements of the spatial profile and temporal evolution of the Ar I 750.4 nm emission line from a 33.3 Pa argon plasma at (a) 75 V, (b) 100 V, (c) 150 V, and (d) 200 V applied rf voltages.

**Fig. 7:**
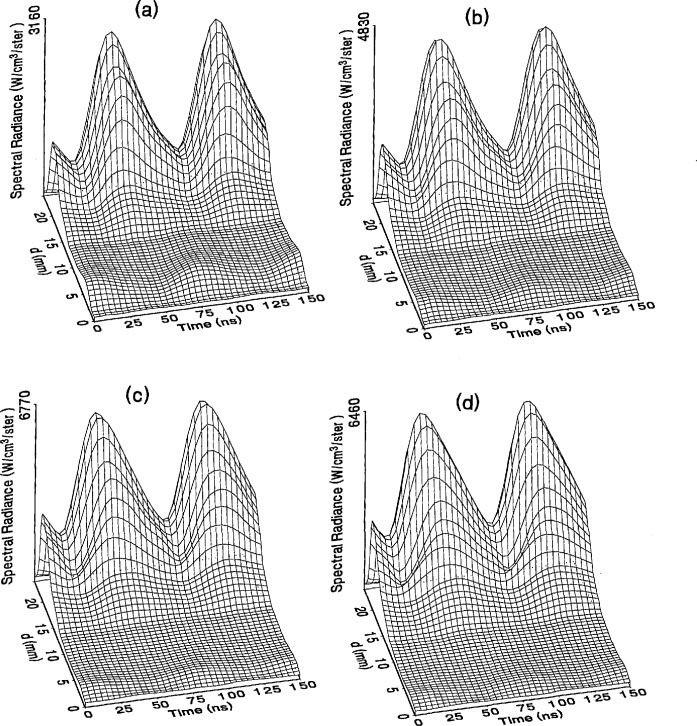
Optical emission measurements of the spatial profile and temporal evolution of the Ar I 750.4 nm emission line from a 66.7 Pa argon plasma at (a) 75 V, (b) 100 V, (c) 150 V, and (d) 200 V applied rf voltages.

**Fig. 8:**
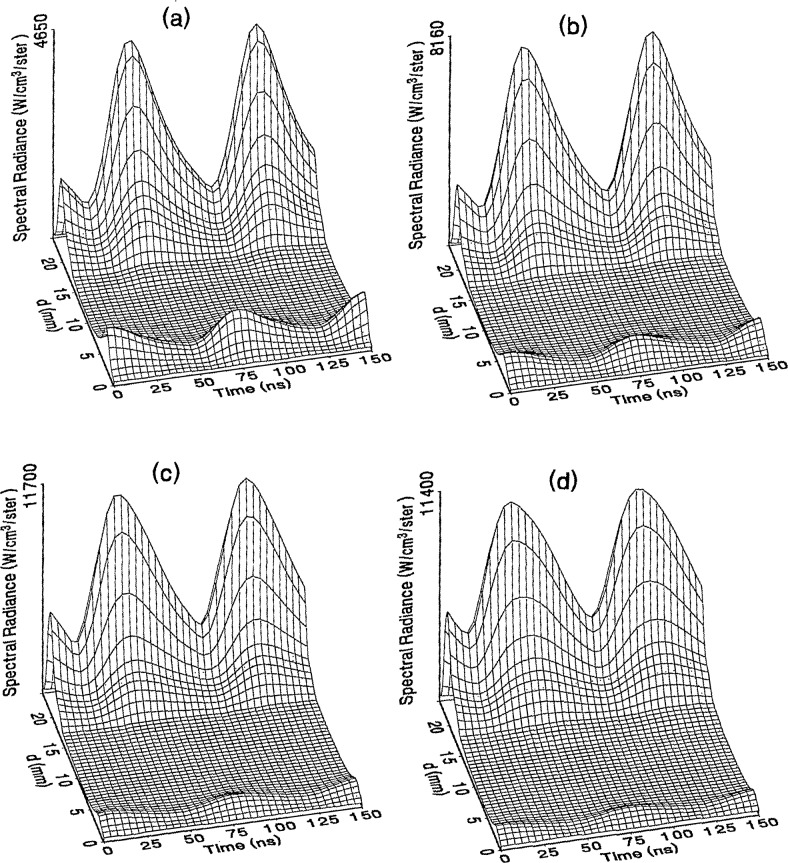
Optical emission measurements of the spatial profile and temporal evolution of the Ar I 750.4 nm emission line from a 133 3 Pa argon plasma at (a) 75 V, (b) 100 V, (c) 150 V, and (d) 200 V applied rf voltages.

**Fig. 9:**
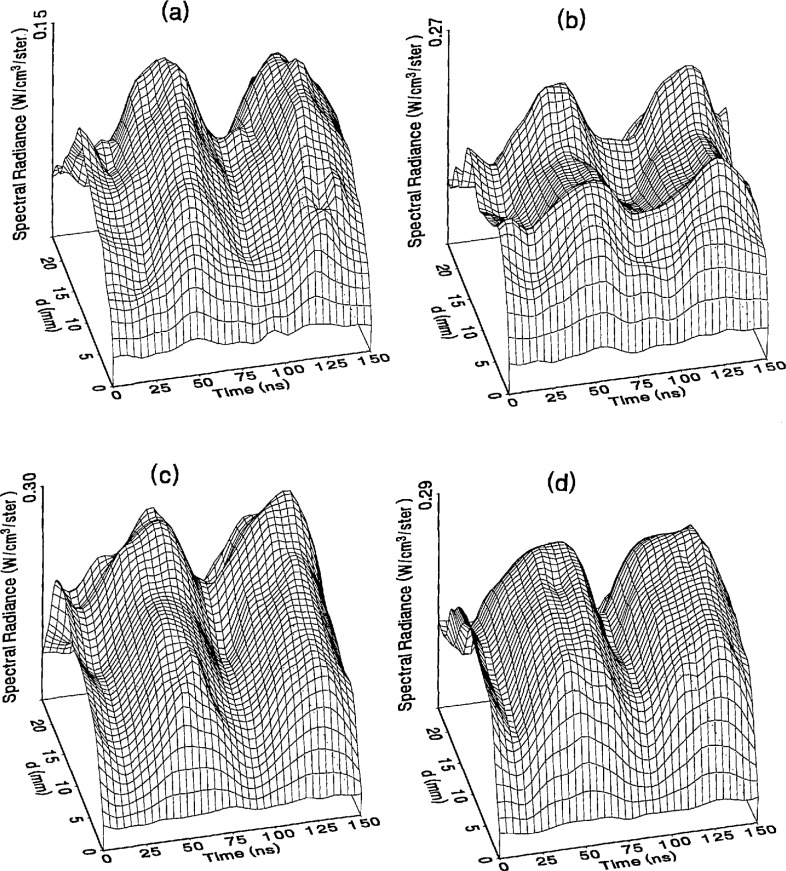
Optical emission measurements of the spatial profile and temporal evolution of the Ar II 434.8 nm emission line from a 6.7 Pa argon plasma at (a) 75 V, (b) 100 V, (c) 150 V, and (d) 200 V applied rf voltages.

**Fig. 10:**
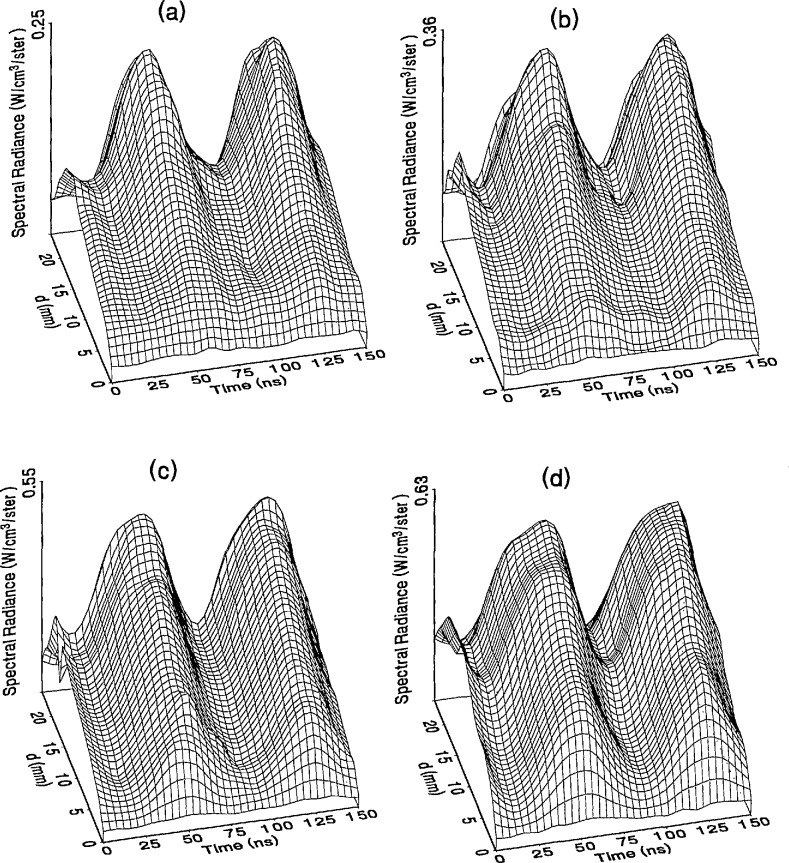
Optical emission measurements of the spatial profile and temporal evolution of the Ar II 434.8 nm emission line from a 13.3 Pa argon plasma at (a) 75 V, (b) 100 V, (c) 150 V, and (d) 200 V applied rf voltages.

**Fig. 11:**
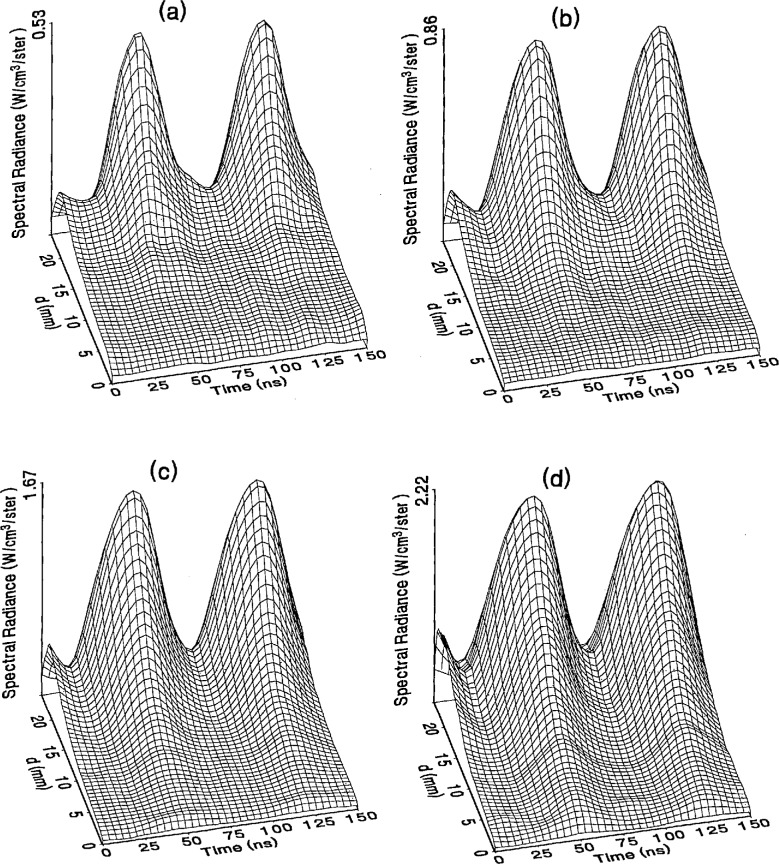
Optical emission measurements of the spatial profile and temporal evolution of the Ar II 434.8 nm emission line from a 33.3 Pa argon plasma at (a) 75 V, (b) 100 V, (c) 150 V, and (d) 200 V applied rf voltages.

**Fig. 12:**
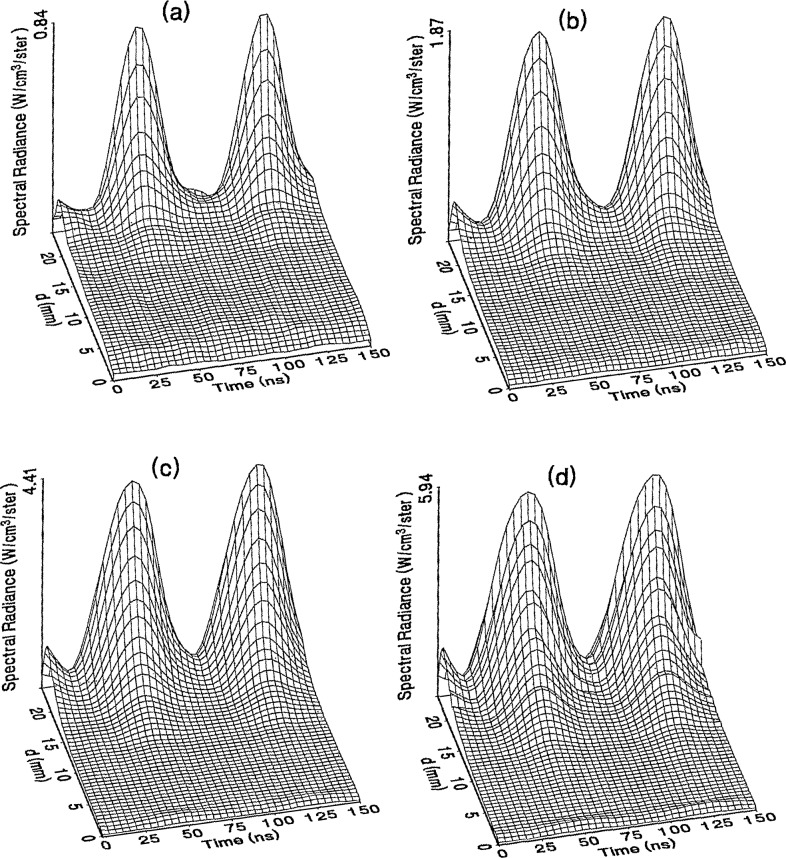
Optical emission measurements of the spatial profile and temporal evolution of the Ar II 434.8 nm emission line from a 66.7 Pa argon plasma at (a) 75 V, (b) 100 V, (c) 150 V, and (d) 200 V applied rf voltages.

**Fig. 13:**
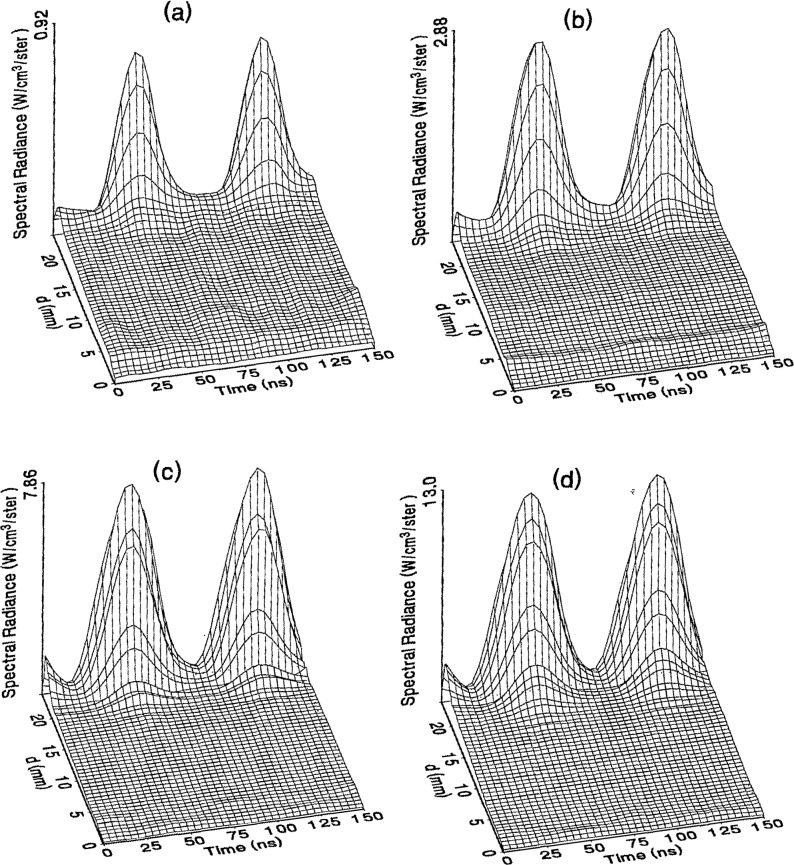
Optical emission measurements of the spatial profile and temporal evolution of the Ar II 434.8 nm emission line from a 133.3 Pa argon plasma at (a) 75 V, (b) 100 V, (c) 150 V, and (d) 200 V applied rf voltages.

The optical emission signals as a function of observation point and time are shown for a range of plasma conditions in Figs. 4–8 for the Ar I line (750.4 nm) and in Figs. 9–13 for the Ar II line (434.8 nm). Data was taken for argon pressures of 6.7, 13.3, 33.3, 66.7, and 133.3 Pa (50, 100, 250, 500, and 1000 mTorr), and uncorrected applied peak-to-peak rf voltages of 75, 100, 150, and 200 V. Figs. 4–13 are plots of surface fits to the calibrated optical emission time profiles taken at many observation points between the electrodes. A time range of 150 ns is shown (~ 2 complete rf cycles), with each square of the grid corresponding to approximately 4 ns on the time axis and 0.5 mm on the *d* axis. The time *t* = 0 was chosen to correspond to the maximum of the voltage waveform. For these figures and for the remainder of this paper *d* is defined as the position of the observation point as measured from the grounded electrode along an axis through the center of the rf electrodes. The vertical axis on each plot is the absolute spectral radiance measured from the discharge. The number at the top of the vertical axis is the magnitude of the maximum observed signal.

Two complete cycles are shown in each plot to help in determining the significance of minor features in the surface plots. If a feature is not repeated in both cycles, then it is not reproducible and should not be considered to be real. This is particularly evident for the low-pressure Ar II data shown in Figs. 9 and 10 where the signal levels were low, resulting in noisy data.

In order to characterize the conditions of the plasmas for which the data in Figs. 4–13 were obtained, data from the measurements of the current and voltage waveforms are presented in Table 1. Listed are the magnitudes and relative phases of the first three Fourier components of the voltage and current waveforms at the surface of the powered electrode, *V_p_*(*t*) and *I_p_*(*t*). Higher order components were too small to be reproducibly measured. For ease of comparison, the time origin of the waveforms was shifted so that in each case the phase of the fundamental current component (*n* = 1) was equal to zero.

## 4. Discussion

As is evident from Figs. 4–13, the temporal and spatial profiles of the optical emission are highly dependent upon the discharge pressure. At 6.7 Pa (Figs. 4 and 9) the spatial profiles are broad with the emission essentially extending across the entire region between the electrodes. Only a small fraction of the optical emission signal exhibits time-dependent behavior at these lower pressures. As the pressure increases to 133.3 Pa the absolute intensity of the optical emission signals increase by a factor 8 for the Ar I line and a factor of 45 for the Ar II line for an applied voltage of 200 V. At these higher pressures a larger fraction of the optical signal exhibits a time dependence, which is in agreement with previous results by Seeböck and Köhler [20] for lower frequency discharges.

**Fig. 14:**
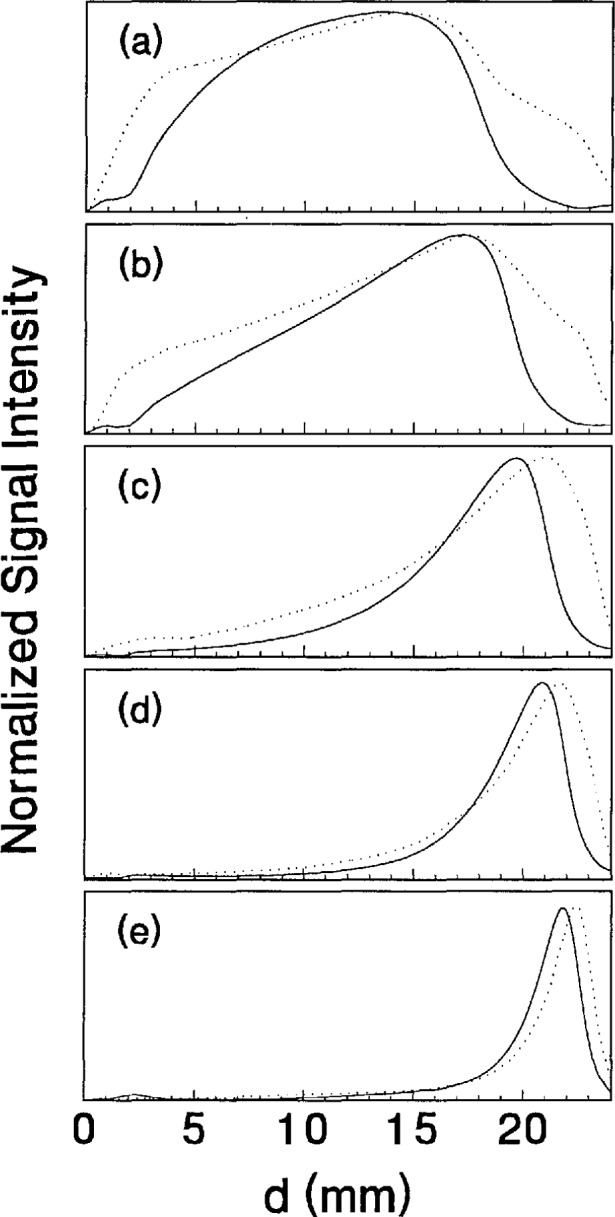
Normalized, time-averaged optical emission spatial profiles taken along the eentral axis of the electrodes for Ar I 750.4 nm (−) and Ar II 434.8 nm (…) lines from argon plasmas. The applied rf voltage was 200 V and the gas pressures were (a) 6.7 Pa, (b) 13.3 Pa, (e) 33.3 Pa, (d) 66.7 Pa, and (e) 133.3 Pa. The position *d* = 0 corresponds to the surface of the grounded electrode.

As the pressure is increased from 6.7 to 133.3 Pa, the most intense regions of emission shift toward the powered electrode and the formation of well-defined sheaths (dark zones near the electrode surfaces) are observed. The formation of sheaths and a bright band of emission near the powered electrode is not due solely to an increase in signal intensity in that region, but also to a decrease in emission intensity in the central regions of the plasma. Careful analysis of the spatial variation in the optical signals indicates that for a given pressure and voltage, this bright band of emission peaks significantly closer to the powered electrode in the Ar II data than for the Ar I emission. This is more evident in Fig. 14 where the time-averaged spatial dependence of the Ar I and Ar II lines are shown for a 200V plasma. Figure 14 also indicates that for most plasma conditions the Ar II emission is spatially broader than the Ar I profile, with the Ar II emission extending closer to both electrodes.

Formation of bright bands near the grounded electrode is observed for only a few sets of plasma conditions. For the Ar I transition, a weak sheath is observed at 66.7 Pa with well defined sheaths present at 133.3 Pa (see Figs. 7 and 8). As expected, the time dependence of the bright bands in front of the grounded electrode is 180° out of phase with the emission signal near the powered electrode. Interestingly, as the applied voltage increases (see Fig. 8) the amplitude of the emission near the grounded electrode remains nearly constant while the emission near the powered electrode increases by a factor of 2.5. This may be related to the fact that the voltage across the sheath near the grounded electrode is nearly independent of the applied rf voltage as shown by electrical measurements [16] and ion kinetic-energy measurements [14].

The existence of a bright band of Ar II emission near the grounded electrode was only observed for high-pressure, high-voltage plasmas (e.g., 200 V, 66.7 Pa). For these conditions a weak increase in the optical signal is detected near the grounded electrode. Unlike the Ar I signal this bright band increases in intensity with increasing voltage, and the time dependence is in phase with the optical signal near the powered electrode.

The changes in the temporal and spatial distributions with applied rf voltage are less dramatic than those observed with changing pressure. As the applied voltage increases, the peak emission intensity increases, particularly at the higher pressures. Additionally, the widths of the peaks in the time dependent signal increase with increasing voltage. This indicates that conditions are favorable for the excitation of the required transitions for emission during a greater portion of the rf cycle at higher applied voltages.

**Fig. 15:**
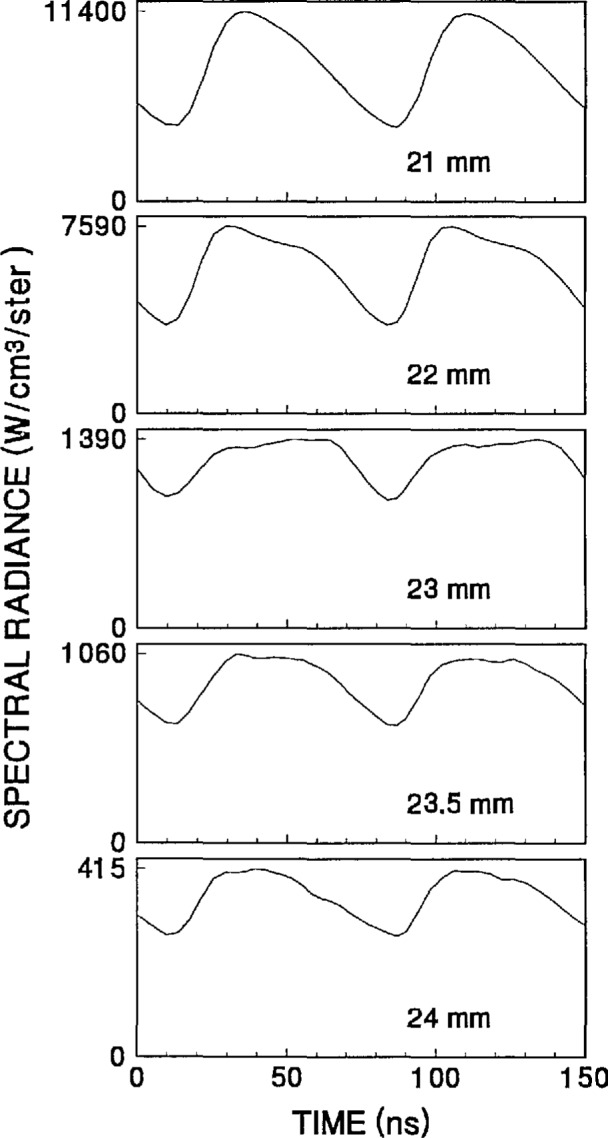
Temporal distributions of the optical emission for the Ar I 750.4 nm line for locations near the powered electrode in a 200 V, 133.3 Pa argon plasma. Distances are given from the grounded electrode.

**Fig. 16:**
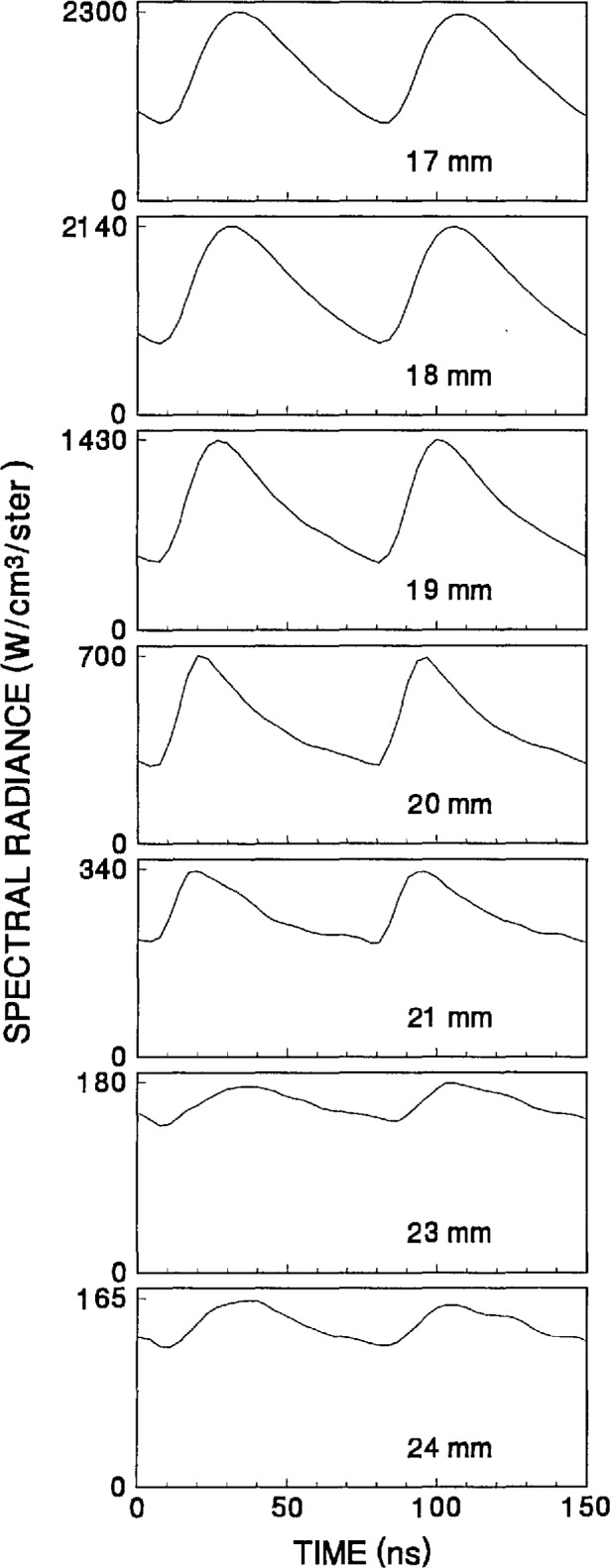
Temporal distributions of the optical emission for the Ar I 750.4 nm line for locations near the powered electrode in a 200 V, 13.3 Pa argon plasma. Distances are given from the grounded electrode.

The time-dependent optical emission signals exhibit very strong spatial dependences near the powered electrode. Figures 15 and 16 show examples of how the shape of the temporal evolution for the Ar I line changes with position in the sheath region of 200 V argon plasmas with pressures of 133.3 and 13.3 Pa, respectively. This type of variance is not observable in Figs. 4–13 due to the viewing angle chosen for the 3-dimensional surface plots, but exists for many of the plasma conditions. The dependence of these temporal distributions on position in the sheath may provide a powerful diagnostic technique for the investigation of sheath dynamics.

## 5. Conclusions

Spatially- and temporally-resolved optical emission profiles have been measured for argon plasmas with pressures ranging from 6.7 to 133.3 Pa, and applied rf voltages from 75 to 200 V. These data include the entire set of “standard” GEC reference cell operating conditions [11] in order to allow for easy comparison with other reference cell measurements. A tabulated set of the optical emission data is available on diskette upon request [21].

The magnitude of the plasma emission for two lines has been put on an absolute scale by comparison with emission from a standard lamp. This allows for a complete characterization of the optical emission signal from the rf plasma, and provides a more comprehensive data set for comparison with theoretical modeling results. A complete set of electrical measurements for all plasma conditions is also provided in order to more fully characterize the discharge.

## Figures and Tables

**Table 1:** Voltage and current parameters for the voltage and current waveforms at the surface of the powered electrode for all plasma conditions used in this paper^a^

Pressure	*V*_rf_	*V*_dc_	V_1_	*V*_2_	*V*_3_	*I*_1_	*I*_2_	*I*_3_	*ϕ_i_*_1_	*ϕ_i_*_2_	*ϕ_i_*_3_	*ϕ_v_*_1_	*ϕ_v_*_2_	*ϕ_v_*_3_	Power
(Pa)	(V)	(V)	(V)	(V)	(V)	(mA)	(mA)	(mA)	(deg)	(deg)	(deg)	(deg)	(deg)	(deg)	(W)
6.7	75	−22.4	42.8	0.3	0.3	65	21	14	0	−104.4	159.2	−67.6	−14.7	−107.8	0.5
	100	−36.0	57.5	0.5	0.6	88	31	25	0	−104.2	174.3	−71.2	−20.0	−93.1	0.8
	150	−65.1	85.1	1.0	1.1	126	57	46	0	−93.9	162.8	−74.4	2.4	−106.7	1.4
	200	−95.0	114.8	0.9	1.5	159	65	61	0	−95.6	−179.3	−76.3	−6.6	−89.3	2.2
13.3	75	−19.0	43.2	0.4	0.4	83	26	17	0	−103.2	146.7	−68.8	−10.0	−119.8	0.7
	100	−35.8	59.3	0.6	0.8	121	41	32	0	−101.2	162.8	−73.2	−8.4	−105.3	1.0
	150	−62.7	85.8	0.9	0.9	172	64	40	0	−89.4	162.0	−76.0	18.6	−106.8	1.8
	200	−94.4	116.6	1.2	1.5	208	84	65	0	−93.5	166.7	−77.5	−3.0	−104.0	2.6
33.3	75	−15.8	43.9	0.4	0.3	124	29	13	0	−107.4	136.1	−68.9	−4.3	−131.1	1.0
	100	−28.1	58.9	0.8	0.6	176	54	26	0	−105.5	144.9	−72.5	−11.1	−122.0	1.6
	150	−58.6	87.5	1.5	1.4	288	103	59	0	−97.7	162.3	−76.8	−1.5	−106.2	2.9
	200	−82.8	119.7	2.9	2.5	558	197	108	0	−94.4	171.0	−78.6	−1.1	−99.1	7.2
66.7	75	−13.5	44.0	0.5	0.4	153	34	14	0	−117.8	126.5	−67.2	−25.5	−141.0	1.3
	100	−26.2	60.6	1.0	0.7	251	66	29	0	−104.2	142.0	−71.4	−2.0	−127.4	2.4
	150	−55.2	89.0	2.2	1.9	429	144	81	0	−97.2	156.1	−75.7	0.9	−113.5	4.7
	200	−82.8	119.7	2.9	2.5	558	197	108	0	−94.3	160.1	−77.6	0.2	−109.1	7.2
133.3	75	−11.9	44.5	0.5	0.2	184	33	10	0	−122.2	130.2	−65.9	−28.2	−138.5	1.7
	100	−25.1	60.5	1.1	0.7	315	77	28	0	−110.9	141.9	−70.4	−18.5	−127.6	3.2
	150	−49.4	89.0	1.8	1.7	524	144	70	0	−102.3	160.7	−73.3	−4.9	−108.4	6.7
	200	−75.2	119.5	3.1	3.0	754	232	125	0	−97.2	163.8	−74.4	−1.9	−106.7	12.1

a *V*_rf_ is the applied peak-to-peak rf voltage, *V*_dc_ is the self-bias potential, *V*_n_ is the magnitude of the *n*th Fourier component of the voltage waveform, *I*_n_ is the magnitude of the *n*th Fourier component of the current waveform, *ϕ_in_* and *ϕ_vn_* are the phases of the *n*th Fourier current and voltage components with respect to *I*_1_, and the power is the power dissipated in the plasma as calculated from the measured waveforms. Therefore the *n*th voltage component is defined by *V_n_* cos (*nωt* + *ϕ_vn_*) and the *n*th current components is *I_n_* cos (*nωt* + *ϕ_in_*) where *ω* is the fundamental angular frequency. The time origin of each waveform has been shifted so that *ϕ_i_*_1_ = 0.

## Footnotes

2 Certain commercial equipment, instruments, or materials are identified in this paper to specify adequately the experimental procedure. Such identification does not imply recommendation or endorsement by the National Institute of Standards and Technology, nor does it imply that the materials or equipment identified are necessarily the best available for the purpose.
